# *Paracoccidioides brasiliensis *pancreatic destruction in *Calomys callosus *experimentally infected

**DOI:** 10.1186/1471-2180-9-84

**Published:** 2009-05-07

**Authors:** Rogério M Fortes, André Kipnis, Ana Paula Junqueira-Kipnis

**Affiliations:** 1 Laboratório de Imunopatologia das Doenças Infecciosas, Departamento de Microbiologia, Imunologia, Parasitologia e Patologia, Instituto de Patologia Tropical e Saúde Pública, Universidade Federal de Goiás, (Research Center for Tropical Diseases, Federal University of Goias) Goiania, Goiás, Brasil

## Abstract

**Background:**

The wild rodent *Calomys callosus *is notably resistant to *Trypanosoma cruzi *infection. In order to better characterize this animal model for experimental infections, we inoculated *C*. *callosus *intraperitoneally with *Paracoccidioides brasiliensis*, a thermally dimorphic fungus that causes a chronic disease with severe granuloma formation in the mouse and humans. The dissemination of *P. brasiliensis *cells through the lungs, liver, pancreas, and spleen was assessed by histological analysis.

**Results:**

The animals were susceptible to infection and showed a granulomatous reaction. *C. callosus *presented peritonitis characterized by the presence of exudates containing a large number of yeast cells. Extensive accumulation of yeast cells with intense destruction of the parenchyma was observed in the pancreas, which reduced the glucose levels of infected animals. These lesions were regressive in the liver, spleen, and lungs until complete recovery. The role of estrogen during *C. callosus *infection with *P. brasiliensis *was addressed by infecting ovariectomized animals. It was observed a reduced inflammatory response as well as reduced extension of tissue damage. Removal of ovaries reestablished the normal glucose levels during infection.

**Conclusion:**

Taken together, the results presented here reveal the pancreas as being an important organ for the persistence of *P. brasiliensis *during infection of *C. callosus *and that estrogen plays an important role in the susceptibility of the animals to this pathogen.

## Background

*Calomys callosus *(Rodentia-Cricetidae), a wild rodent, exists near farm residences in savannas and cattle breeding areas. It has been adapted to be bred in captivity under controlled laboratory conditions and values for reproductive parameters, such as age at reproduction, pregnancy time, number of litters, male/female ratio, growth curve, and some external anatomical values have also been determined [1,2]. Laboratory inbred strain was obtained for experimental purpose [3,4]. This rodent has been described as a reservoir of *Trypanosoma cruzi*, the causative agent of Chagas disease and of the hantaviroses, zoonoses caused by the Bunyaviridae family [5,6].

*C. callosus *naturally and experimentally infected with *T. cruzi *presents high parasitaemia values during the presumable first days of infection, which progressively decreases until becoming negative a few weeks later showing regression of the lesions within a few days [7]. The infection is accompanied by inflammation of both myocardium and skeletal muscle characterized initially by an infiltrate containing macrophages, fibroblasts and small numbers of lymphocytes. Although the mechanism underlying the resistance of *C. callosus *to *T. cruzi *infection is not totally understood, its ability to control and avoid tissue lesions might be a key factor involved in its resistance to pathogens [5,6,8,9]. Nevertheless, when *C. callosus *was experimentally infected with *Toxoplama gondii*, they were highly susceptible and all animals died on the acute phase of the infection [10].

*Paracoccidioides brasiliensis *is a thermally dimorphic fungus that causes a chronic disease with severe granuloma formation widely spread in Latin America [11]. Different *P. brasiliensis *strains have been evaluated in the mouse model of infection showing notably differences in the susceptibility pattern [12,13]. Because of the unique response of *C. callosus *to different pathogens they may be useful as an animal model for the development of experimental infections by *P. brasiliensis*. A recent work showed that *C. callosus *succumbs to the *P. brasiliensis *strain 18 infection, presenting evidence of inflammatory reaction in several organs and specific humoral response to *P. brasiliensis *antigens [14]. Natural infection of *C. callosus *with *P. brasiliensis *has not yet been reported even though they reside in endemic areas of Paracoccidioidomycosis (PCM). The mechanisms underlining the protective immune response for PCM seems to involve estrogen, because women tend to be more resistant to the infection, added to the fact that estrogen avoids the transition from conidia to yeast, the infective form of infection [11,15].

A *P. brasiliensis *strain isolated from a patient in the Brazilian savannas (PB01) was shown to be more virulent than the strain 18 [16]. This study was designed to analyze the infection of *C. callosus *with PB01 strain by investigating the inflammatory lesions in several organs as well as to investigate the role of estrogen in the susceptibility of the animals. In order to evaluate whether estrogen affects the *C. callosus *susceptibility, the ovaries were removed because they are the main source of estrogen.

In this report we present data supporting the susceptibility of *C. callosus *to infection with PB01 strain, which is resolved after 90 days in the liver, lungs, and spleen, but viable fungi remained during all studied time in the pancreas. We also demonstrate that the persistence of the fungus in the pancreas alters glucose levels. Evidence is shown about the involvement of estrogen in the inflammatory response.

## Methods

### Fungal suspensions and growth conditions

*Paracoccidioides brasiliensis*, strain 01 was provided by the Mycology collection of Research Center for Tropical Pathology – Federal University of Goiás. The yeast forms were grown on solid Fava Netto agar medium at 37°C. After 7 days, the yeast cells were harvested, washed in sterile saline, and adjusted to 10^8 ^cells/mL based on haemocytometer counts. Viability, determined by the fluorescein and ethidium bromide staining methods, was always higher than 85% [17].

### Animals

Adult female *C. callosus *(8–12 weeks) were used throughout this study. The animals were bred in the Animal Facilities of the University of São Paulo and Research Center for Tropical Pathology – Federal University of Goiás. The animals were handled and maintained under strict ethical conditions according to the international recommendations for animal welfare (NIH Publication n° 23, 1985) and the Ethical Committee for human and animal sciences of Federal University of Goiás (012/1999).

### Experimental procedures

In order to evaluate the blood leukocyte and glucose levels of *C. callosus *infected with *P. brasiliensis*, the animals were i.p. injected followed by macroscopic and microscopic evaluations done at days 7, 15, 30, 45, 60, and 75 post infection (three to four animals were analyzed per group at each time point of infection). The organs showing macroscopic lesions were selected for further analysis. Control groups consisted of three animals per time point inoculated with sterile saline.

To determine the role of estrogen during *P. brasiliensis *infection, an additional *C. callosus *group (seventy animals) was subdivided into two sets: one being bilaterally ovarectomized (31 animals) and the other sham-operated (39 animals). Forty days after surgery, all animals were inoculated in the peritoneum with 1 × 10^6 ^viable infective forms of *P. brasiliensis*. An additional control group consisting of non-operated and non-infected animals (5 animals per time point) received only saline injection.

### Histology

On days 15, 45, 60, and 75 of infection, two to three animals from each group were sacrificed, grossly inspected, and fragments of mesentery, liver, spleen, pancreas, and lungs were collected and fixed in 10% formaldehyde. Representative sections from each organ were embedded in paraffin, processed and stained with haematoxilin-eosin (HE). Quantification of the lesion extensions was determined using a computer-aided densitometric software (OPTIMAS Bioscan Inc. WA, USA). For each organ, five slides with tissue sections were entirely evaluated. The number and area of the granulomas were determined, and the extent of tissue section occupied by the lesion was calculated by dividing the area occupied with lesions by the total area of the organ.

### Leukocyte counts and glucose levels

Blood samples for leukocyte counts or glucose determinations were withdrawn from the retro-orbital plexus. Leucocytes were counted in a haemocytometer and the results were reported as number of leukocytes per mL of blood. Serum glucose levels were determined by the method of Trinder [18] and reported as mg/dL.

## Results

### PB01 infection in *Calomys callosus*

**Figure 1:**
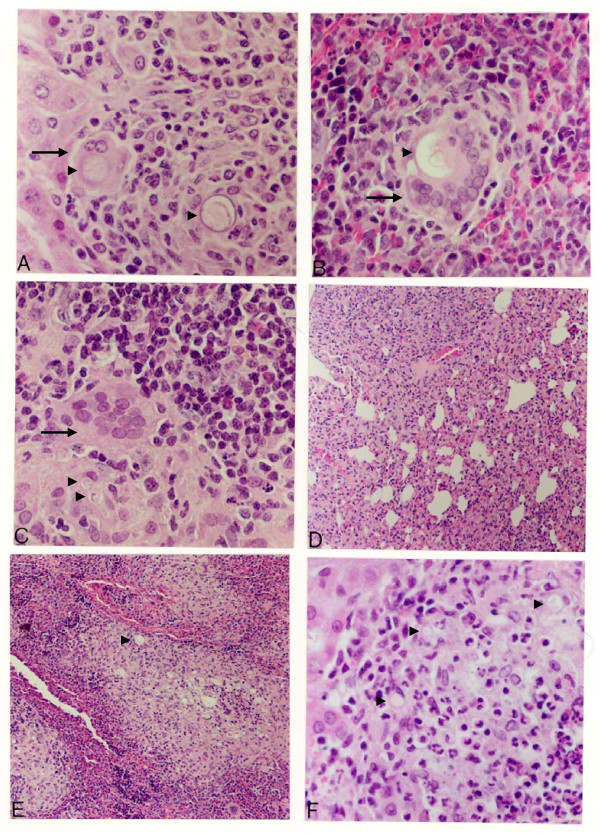
**Histological findings during the infection of *C. callosus *with *P. brasiliensis***. The tissue sections of liver, pancreas, lung, spleen and lymph nodes were stained with haematoxylin-eosin and examined at 200× (A, B, C and F) or 100× (D and E) magnification. In A and B, liver and spleen 15 days post infection, respectively; C and D mesenteric lymph nodes and lung 45 days post infection, respectively; and in E and F, spleen and liver at 75 days post infection. Fungi cells are pointed with arrowheads. Giant cells are pointed with arrows.

**Figure 2:**
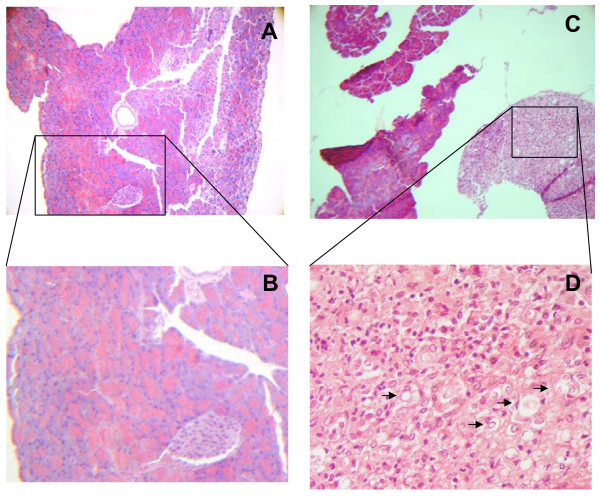
***C. callosus *pancreas histological findings 75 days post infection with *P. brasiliensis***. Fungi cells are pointed with arrowheads.

Gross inspection of *C. callosus *i.p. infected with 10^6 ^yeast forms of PB01 revealed peritonitis characterized by the presence of exudates containing a large number of yeast cells. Adherence involving several parts of mesentery and spleen was also observed. These signs increased in intensity with time from injection of the fungus until the infection turned to the chronic phase (sixty days post infection). Following the acute phase of the inflammatory reaction, the infection became circumscribed due to granuloma formation in the peritoneal cavity as well as in several distant organs such as the liver, spleen, lungs, and pancreas. Histopathological analysis on day 15 of infection showed typical granulomas that had appeared in the liver and spleen. In the liver, giant cells containing phagocytosed yeast cells were surrounded by a lymphocyte and monocyte (macrophage) – rich cell infiltrate with some scattered polymorphonuclear leukocytes (Fig. 1A). In the spleen, granulomas were more organized, presenting an outer mantle of histiocytes, and giant cells also containing yeast (Fig. 1B). Later, on the 45^th ^day of infection, granulomas were also found in the mesenteric lymph nodes. Although giant cells and histiocytes were present in those organs, typical forms of the yeast were not detected (Fig. 1C). In the lungs, an interstitial inflammation without the presence of granulomas was observed. Lymphocytes, histiocytes, and polymorphonuclear leukocytes were found all over the parenchyma (Fig. 1D). After 75 days of infection, the granulomas originally observed in the spleen and liver (Fig. 1E and 1F, respectively) became disorganized. Degenerated yeast cells were found inside necrotic areas usually containing large number of polymorphonuclear leukocytes (Fig. 1F). Extensive accumulation of live yeast cells with intense destruction of the parenchyma was observed in the pancreas after 80 days of infection (Fig. 2).

**Figure 3:**
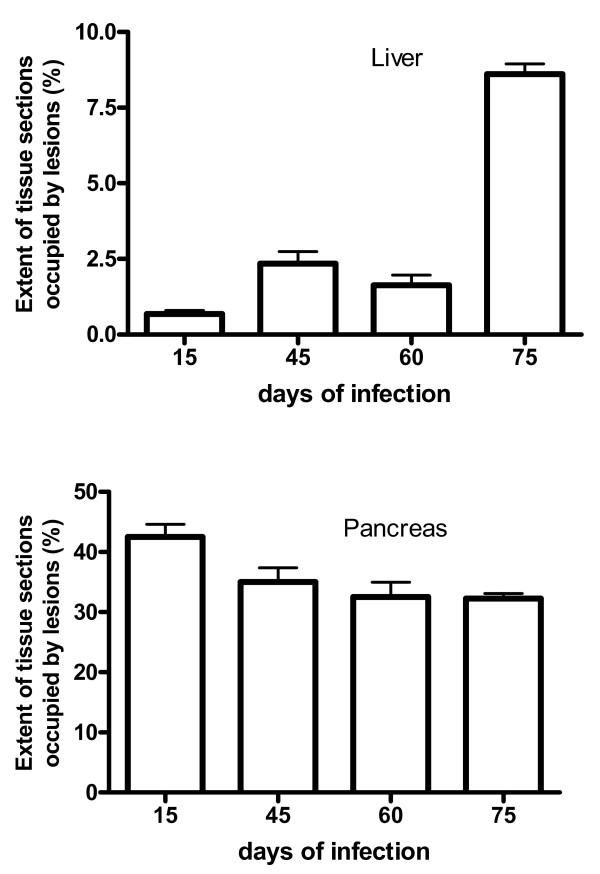
**Extension of tissue sections occupied by the lesions induced by *Paracoccidioides brasiliensis *infection in the liver (A) and pancreas (B) of *Calomys callosus *expressed as percentage**. The results were obtained with the Optimas software. Each bar represents the mean + sd of 5 animals per group.

In order to enumerate the pancreas and liver areas occupied by lesions, the organs were measured and the percentages of lesions were determined. Fig. 3 shows the percentages of the areas taken by the lesions in infected animals. The liver presented a smaller extension of tissue occupation by the lesion that progressively increased but never exceeded 10% of the organ. In contrast, the pancreas showed larger extensions of areas occupied by lesions (greater than 25%) that were maintained through out the study.

**Figure 4:**
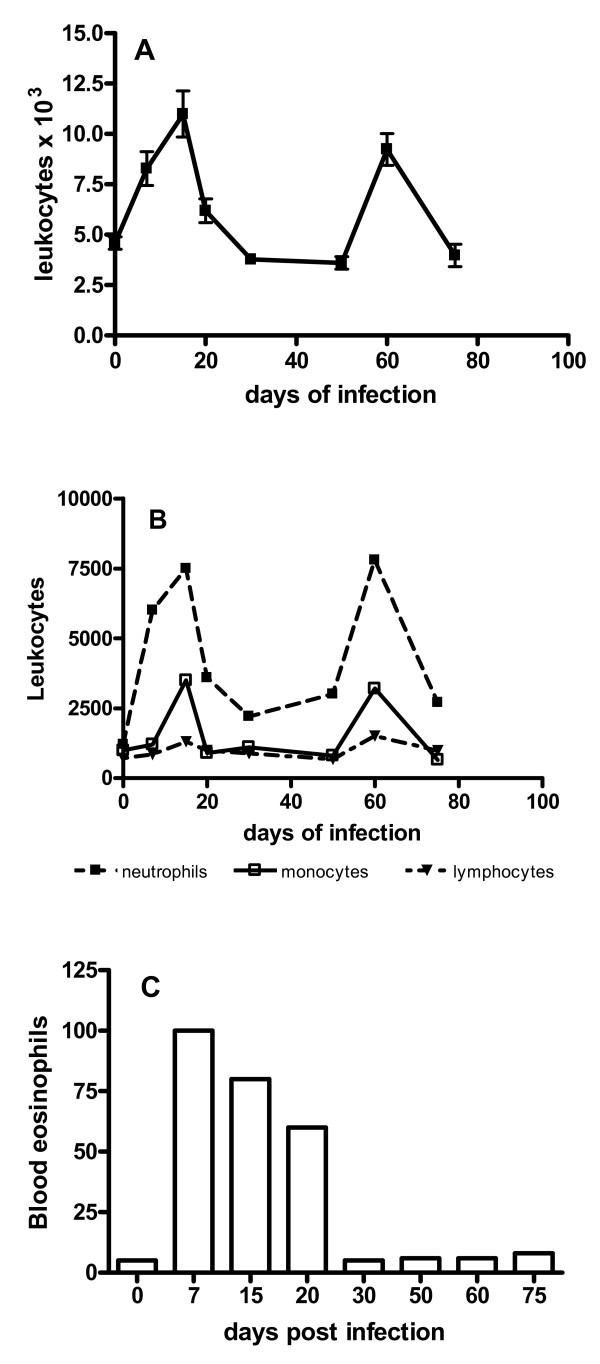
**Leukocyte levels in the blood of *Calomys callosus *during infection with *Paracoccidioides brasiliensis***. A – Each point represents the mean ± standard deviation of counts of total leukocytes in blood samples from 4 animals. B – Absolute numbers of neutrophils, lymphocytes, and monocytes. C – Absolute numbers of eosinophils.

The recruitment of leukocytes from bone marrow to the blood is a good parameter to evaluate the general infection status of the animal and to predict the prognosis of the infection. *C. callosus *injected with 1 × 10^6 ^yeast forms of *P. brasiliensis *presented leukocytosis at days 20 and 60 after infection (Fig. 4A). On the 20^th ^day of infection, lymphocytes and neutrophils were the predominant cells whereas on the subsequent days, although lymphocytes remained the major cell population, monocytes surpassed neutrophils (Fig. 4B). A peak of eosinophil numbers was detected on the 20^th ^day, progressively decaying thereafter.

### Effect of *P. brasiliensis *infection on glucose blood levels of *C. callosus*

**Figure 5:**
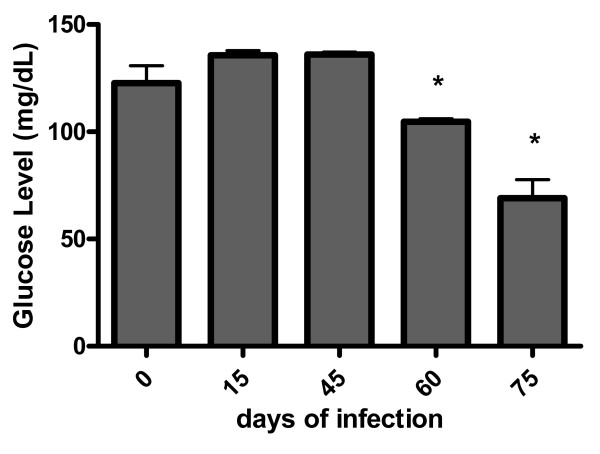
**Serum glucose in *Calomys callosus *during infection with 1 × 10^6 ^yeast forms of *Paracoccidioides brasiliensis***. Bars represent the mean and standard deviation of 4–5 animals per group. * Statistically different from controls, ANOVA, T test, p < 0.05.

Based on the observations that the pancreas was seriously compromised throughout infection, we questioned whether this fact could affect the serological glucose levels of *C. callosus*. As shown in Fig. 5, infected animals start to loose control of glucose levels after 60 days of infection, when serum levels drop as the infection progresses.

### Effect of ovariectomy on *P. brasiliensis *infection of *C. callosus*

**Figure 6:**
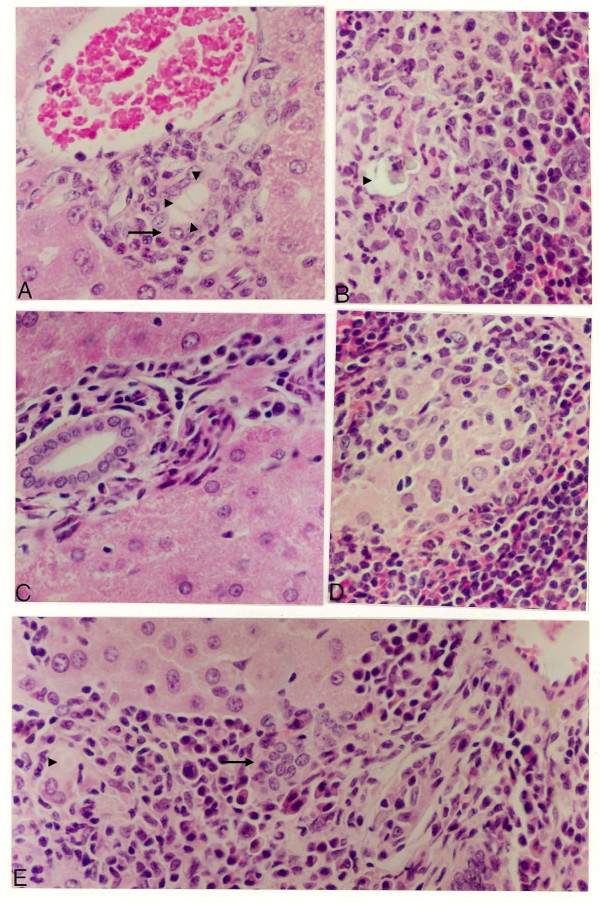
**Histological analyses of female *Calomys callosus *infected i.p. with *Paracoccidioides brasiliensis *after bilateral ovariectomy**. The tissue sections stained with haematoxylin-eosin were examined at a magnification of 200 X. In A and B – liver and spleen of animals 15 days post infection, C and D – liver and spleen 45 days post infection; E – liver 75 days post infection. Dead fungi cells are pointed with arrowheads. Giant cells are pointed with arrows.

It has been shown that estrogen hormone is one of the *P. brasilensis *infection resistance mechanisms [19]. In order to understand the estrogen role in the *C. callosus *infection, infected ovariectomized animals were compared to sham-operated animals. The infection progression in sham-operated animals developed similarly as in non-operated animals (Fig. 1 and data not shown). The lesions observed in ovariectomized animals showed that the infiltrate contained fewer inflammatory cells and that the parenchyma of the liver (Fig. 6A, C and 6E) and spleen (Fig. 6B and 6D) were damaged. The inflammatory lesions seen in the liver of ovariectomized animals were concentrated in the space of Dissé until the 45^th ^day of infection (Fig. 6A and 6C). A fewer number of yeast debris were observed in ovariectomized infected animals compared to sham-operated infected animals, throughout the study (Fig. 6). At day 75, a diffuse mononuclear infiltration was observed in the liver although with very few intact parasites. As early as 15 days post infection, a neutrophil infiltrate was observed in the spleen (Fig. 6B) that was not seen later on infection (Fig. 6D).

**Figure 7:**
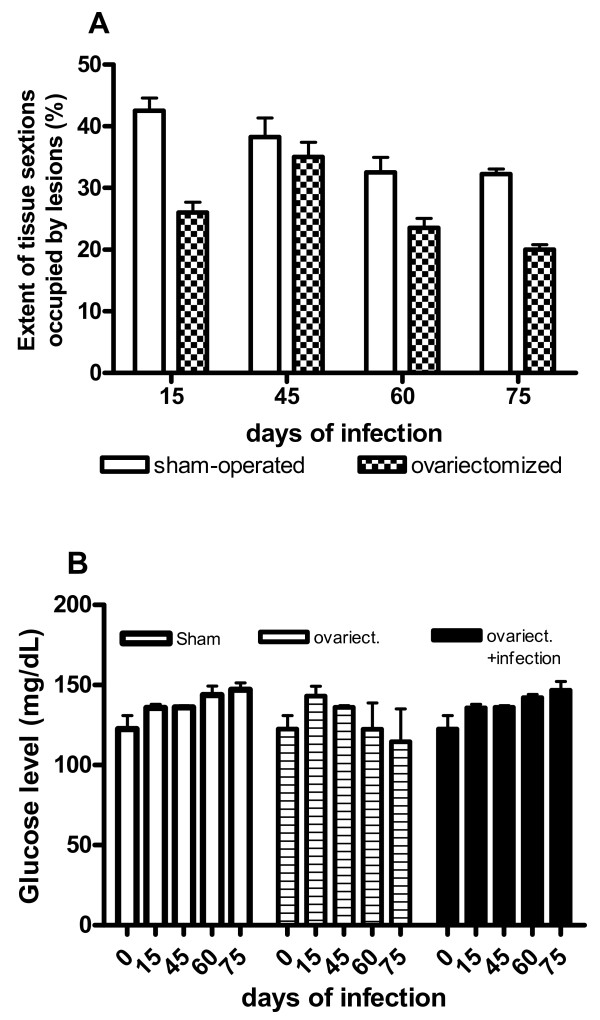
**Effect of the ovariectomy on the tissue extension and glucose serum levels in ovariectomized or sham-operated *Calomys callosus *infected with 1 × 10^6 ^yeast forms of *Paracoccidioides brasiliensis***. A – Extension of tissue sections occupied by the lesions induced by *Paracoccidioides brasiliensis *infection in the pancreas. B – Serum glucose levels. Bars represent the mean and standard deviation of 4–5 animals per group.

As stated above, ovariectomy significantly altered the infection progression in the liver and spleen of infected *C. callosus*, consequently we investigated if the pancreas would be affected by the deprivation of estrogen due to the removal of the ovaries. Surprisingly, there was no significant difference of tissue sections occupied by the lesions in the pancreas between the sham-operated and ovariectomized animals (Fig. 7A). Infection of ovariectomized *C. callosus *prevented the drop of glucose levels seen in sham-operated and infected animals (Fig. 7B).

## Discussion and conclusion

Several species of wild animals are known to harbor many types of infectious agents. The induced infections usually are silent, most likely due to efficient immunologic mechanisms of resistance resulting from years of co-evolution of hosts and pathogens. In nature, armadillos (Dasypus noveminctus) were found infected with *P. brasiliensis *in endemic area [20,21]. *C. callosus *and human beings in endemic area of paracoccidioidomycosis constitute one example in which pathogenic fungus and a regional well established rodent are living in a close environmental relationship. However, there are no reports describing *C. callosus *infected with *P. brasiliensis *in nature. The lack of such information can be alternatively ascribed either to a complete resistance of *C. callosus *to the fungus or to an efficient immune resistance developed by the host. The later hypothesis is however the most probable in face of the demonstration in this present report and by others [14], that this rodent can be experimentally infected with *P. brasiliensis*.

The granuloma formation in PCM varies in humans and experimental animals according to several factors such as inoculum, route of infection, host susceptibility, and resistance. Previously, it was shown that using a virulent *P. brasiliensis *18 strain, *C. callosus *presented a destructive granuloma formation and disease progression [14]. However, that work failed to show the lesion and granuloma formation in several other important organs. The present work demonstrated for the first time that these animals showed a different inflammatory response at the inoculation area (peritoneum and pancreas) compared to disseminated areas (liver and lungs). The granulomatous reaction organized in *C. callosus *infected with *P. brasiliensis *in the spleen, liver, and lungs, was characterized by an inflammatory cell infiltrate rich in epithelioid cells scattered over the lesions, in contrast to the organized granulomas observed in the mouse model of infection [13]. The number of viable fungi diminished quickly in spleen, liver and lungs during the infection until complete disappearance after 60 days of observation. The disagreement between our findings and a recently published data [14] could be attributed to several important factors such as host susceptibility characteristics as a consequence of different *C. callosus *genetic backgrounds, ours being isogenic strains [3]; animal housing conditions; and *P. brasiliensis *strain virulence differences due to a distinct *P. brasiliensis *isolate (PB01), and laboratory culture collection maintenance procedures. Our results are consistent with the pattern of experimental infection of *C. callosus *with *T. cruzi*, where all the infected animals survived but had positive parasitological tests, until the end of the experiments. The lesions induced by this parasite were characterized by severe inflammation in the myocardium and skeletal muscle, which gradually subsided becoming absent or residual on the 64^th ^day of infection [1,6,9,22]. Thus, with two distinct infection agents, *P. brasiliensis *and *T. cruzi, C. callosus*, although able to acquire experimental infections, became cured or without detectable tissue lesions as the time elapsed.

Despite the fact that lungs, liver, and lymph nodes showed no detectable lesions in the chronic phase of infection, *C. callosus *developed persistent pancreatic infection. This observation may be due to the local peritoneal involvement, as a consequence of the inoculation site. Similarly, macroscopical observations revealed that the minor omentum was the most affected tissue by the infection, which is colocalized with the pancreas. These findings prompted us to address the question whether the fungi growth alters the endocrine homeostasis of *C. callosus*. As the infection with *P. brasiliensis *destroys the pancreas, one would expect alterations on glucose serum levels affecting the survival of the animals but, surprisingly, in our experiments *C. callosus *had a long term surviving curve (more than 250 days after the infection, Fig. 2). This hypothesis was confirmed by our results as *C. callosus *infected with *P. brasiliensis *showed a significant reduction of glucose levels as infection progressed (Fig. 3 and 5). Taken together, these data infer that the infection progression develops differently in accordance to the anatomical site, reinforcing that the pancreas could present an adequate environment for the fungi development.

As seen in several infectious disease models, *P. brasiliensis *infection also induces leukocytosis. The leukocytes blood levels were higher during the infection as compared with the non-infected animals (Fig. 4A, day 0). *C. callosus *presented two distinct leukocytosis peaks flanked by periods of normal blood cell counts. It is interesting to note that when lymphocytes and monocytes blood levels were higher, the granulomatous reaction unexpectedly presented polymorphonuclear cells (PMC) and mononuclear cells (MNC). An interesting observation was the presence of eosinophils seen in the granulomas and in the blood of infected animals at the early stages, a fact that is not present during infection in the mouse model [4,13]. The presence of eosinophils in the analyzed organs, except the pancreas, correlated positively with parasite clearance. A diverse picture of granulomas was however observed coinciding with the second peak of leukocytosis: high monocyte blood cells counts and predominance of macrophages in the granuloma cell infiltrates. The persistence of the leukocytosis until 105 and 120 days of infection could be ascribed to higher colonization of the pancreas by the fungi, in view of the fact that at the correspondent time the lesions in the others organs had attained complete recovery.

Paracoccidioidomycosis incidence in humans appears to be higher in men than in women [11,15]. This difference being attributed either to inhibition of the conversion of mycelium into yeast forms of growth provoked by estrogen or by non-specific host resistance to the fungus [19]. The analysis of mechanisms underlying estrous cycle and host resistance to *P. brasiliensis *has been reported [19]. Sano et al, [19] showed that even using three different inoculation routes, the clearance of the yeast cells in mice, was influenced by the estrogen presence. All female mice presented lower bacterial burden in the blood, peritoneal cavity, and lungs when compared with males. In order to verify if such gender-determined resistance also occurs in *C. callosus *we investigated the effect of the estrogen using ovariectomized animals to eliminate the source of estrogen. The lesions found in sham-operated and ovariectomized animals were equally occupied by large numbers of the fungi. Despite having the same amount of fungi, the sham-operated group presented a more vigorous liver inflammatory response. We also showed that ovariectomized infected *C. callosus *presented more organized granulomatous lesions with fewer pancreatic lesions. Thus the inflammatory response to *P. brasiliensis *was directly affected by the absence of the estrogen which could be one of the aspects contributing to the susceptibly of the disease.

Although, in ovariectomized animals the lesions in liver, spleen, and lungs rapidly evolved to the reorganization of the organ structures, the fungus progressively colonized the pancreas. The process of pancreas colonization was gradual, occurring in both ovariectomized and sham-operated animals (Fig. 7A). Therefore, it can be suggested that *C. callosus *is capable of sequestering the yeast forms of *P. brasiliensis *in the pancreas allowing their reproduction, without dissemination. The mechanisms underlying such fungus tropism to a particular organ deserve further investigation.

Estrogen hormones positively affect the synthesis and release of insulin by pancreatic cells [23]. The extensive colonization of the pancreas by fungi was expected to disturb the functions controlled by this organ. Indeed sham-operated *C. callosus *presented along the infection reduction of glucose blood levels, when compared with the non-infected sham-operated animals. The decrease in glucose levels was not observed in ovariectomized and infected animals, supporting the protective effect exerted by the absence of the estrogen during infection.

In this study, it was observed: a) The experimental infection of *C. callosus *by *P. brasiliensis *is different from the other animal models since the organized granulomatous lesions are more diffuse and gradually diminished, b) In *C. callosus *the pancreas were persistently infected, c) The function of the pancreas was affected by the infection of *C. callosus*, and d) The presence of estrogen directly affected the pancreas function of infected animals. The results presented here show a predisposition of the *P. brasiliensis *to grow in the pancreas of *C. callosus*.

## Competing interests

The authors declare that they have no competing interests.

## Authors' contributions

RMF carried out the ovariectomy studies, and drafted the manuscript. AK carried out the immunoassays, drafted the manuscript, and participated in the design of the study. APJK conceived the study, performed the statistical analysis, and participated in its design and coordination. All authors read and approved the final manuscript.
